# Challenges and opportunities for modeling monogenic and complex disorders of the human retina via induced pluripotent stem cell technology

**DOI:** 10.1515/medgen-2021-2092

**Published:** 2021-12-03

**Authors:** Karolina Plössl, Andrea Milenkovic, Bernhard H. F. Weber

**Affiliations:** Institute of Human Genetics, University of Regensburg, 93053 Regensburg, Germany; Institute of Clinical Human Genetics, University Hospital Regensburg, 93053 Regensburg, Germany

**Keywords:** retina, complex disorder, monogenetic disease, model system, age-related macular degeneration, bestrophinopathies

## Abstract

The human retina is a highly structured and complex neurosensory tissue central to perceiving and processing visual signals. In a healthy individual, the close interplay between the neuronal retina, the adjacent retinal pigment epithelium and the underlying blood supply, the choriocapillaris, is critical for maintaining eyesight over a lifetime. An impairment of this delicate and metabolically highly active system, caused by genetic alteration, environmental impact or both, results in a multitude of pathological phenotypes of the retina. Understanding and treating these disease processes are motivated by a marked medical need in young as well as in older patients. While naturally occurring or gene-manipulated animal models have been used successfully in ophthalmological research for many years, recent advances in induced pluripotent stem cell technology have opened up new avenues to generate patient-derived retinal model systems. Here, we explore to what extent these cellular models can be useful to mirror human pathologies *in vitro* ultimately allowing to analyze disease mechanisms and testing treatment options in the cell type of interest on an individual patient-specific genetic background.

## Zusammenfassung

Die menschliche Netzhaut ist ein hochstrukturiertes und komplexes neurosensorisches Gewebe, das eine zentrale Rolle in der Wahrnehmung und visuellen Verarbeitung optischer Signale spielt. Im gesunden Individuum ist das enge Zusammenspiel zwischen der neuronalen Netzhaut, dem eng benachbarten retinalen Pigmentepithel und der unmittelbaren Blutversorgung, der Choriokapillaris, kritisch für ein lebenslang intaktes Sehvermögen. Eine Beeinträchtigung dieses empfindlichen und metabolisch hochaktiven Systems, sei es durch genetische Veränderung, individuelle äußere Einflüsse oder beides, führt zu einer Vielfalt von pathologischen Phänotypen der Netzhaut. Verstehen und Behandeln dieser Krankheitsprozesse werden durch einen hohen medizinischen Bedarf an Therapieoptionen bei jungen, aber auch älteren Patienten, motiviert. Während natürlich vorkommende oder Gen-manipulierte Tiermodelle viele Jahre erfolgreich in der Gesundheitsforschung eingesetzt wurden, haben jüngere Entwicklungen im Bereich der induzierten pluripotenten Stammzell-Technologie neue, bisher nicht bekannte Möglichkeiten geschaffen, Patienten-abgeleitete Zellmodelle der Netzhaut zu generieren. In diesem Review möchten wir explorieren, in welchem Maße solche zellulären Modelle hilfreich sein können die menschlichen Pathologien *in vitro* abzubilden und somit deren Krankheitsmechanismen zu verstehen, was wiederum zu Behandlungsoptionen in einem definierten Zelltyp auf einem individuellen Patienten-spezifischen genetischen Hintergrund erlauben würde.

## Introduction

Sensing their immediate surroundings is essential for living organisms. Amongst the five senses of touch, smell, taste, hearing and sight, sight generally is considered the most important and least dispensable skill [1]. Our current knowledge about the structure and function of the neuronal retina, a tissue of utmost importance for maintaining eyesight, was pioneered more than 125 years ago by the Spanish neuroscientist Santiago Ramón y Cajal when he published his first artistic drawings of various retinal cell types [2]. Since then, our knowledge has greatly advanced, providing a molecular framework to understand how a photon is converted to an electrochemical signal which is relayed to the brain and processed to allow an accurate image of our environment.

The human retina consists of more than 50 distinct cell types belonging to functionally related groups such as ganglion cells, amacrine cells, bipolar cells, horizontal cells, and photoreceptors. It is the photoreceptor cells that accomplish the conversion of light to a transmittable electrochemical signal, a process known as phototransduction. While the cone photoreceptors are responsible for high-acuity vision and the perception of color, rods enable low-light vision at dawn or under night sky illumination. To ensure a continuous supply of visual pigment in rod and cone photoreceptors, a highly efficient recycling process takes place in the retinal pigment epithelium (RPE), a postmitotic single-layered polarized cell population localized between the photoreceptors and their steady blood supply, the choriocapillaris [3]. The RPE confers a number of specific functions essential for retinal homeostasis including (i) photoreceptor outer segment (POS) phagocytosis and recycling, (ii) formation of the blood–retina barrier, (iii) stray light absorption, (iv) directed transport of metabolites, nutrients and ions and (v) secretion of cytokines and growth factors (summarized in [4]).

Retinal dystrophies and dystrophies of the RPE represent a heterogenous group of distinct clinical entities with often overlapping symptoms making genetic testing essential to confirm diagnosis. Currently, pathological mutations in almost 300 genes have been reported to be causal for monogenic retinal phenotypes [5]. There is still a major gap between our knowledge of the molecular causes of retinal disease and treatment options which only unfold slowly in recent years. To further intensify the translational process, we need to resolve the cellular mechanisms underlying the retinopathies. A variety of model systems have been applied in the past which were designed to delineate both monogenic and complex retinal degenerations. In vision research, model organisms exist within many phyla whereby some species have become more popular than others, such as fruit flies, frogs, mice, dogs, pigs and non-human primates. Of course, findings made in these diverse organisms have to be considered with care due to often marked anatomical and physiological differences to the human species. Cell culture models, on the other hand, commonly provide only selected features of a disease phenotype and generally fail to account for systemic or paracrine effects driven by cell types not representing the primary site of pathology. Even more importantly, commercially available cell lines often lack important features of the native cells due to bottleneck effects during cell culturing. RPE cell lines are commonly derived from primary (fetal) human RPE cells or represent spontaneously immortalized RPE cells, the latter known as ARPE-19 cells [6]. ARPE-19 cells are widely used in ophthalmic research, while only defined culturing conditions ensure that the lines are not divergent from the original mother cell line in features characteristic for RPE cells *in vivo*, such as pigmentation, polarity and the ability to phagocytose POS [7]. Compounding this problem is the widespread use of ARPE-19 cells in an undifferentiated and possibly unpolarized state to attempt to model RPE functions.

In recent years, induced pluripotent stem cell (iPSC) technology has revolutionized retinal/RPE research by allowing to differentiate many of the retinal cell types of interest from patient-derived iPSCs. This is particularly the case for the RPE-related pathologies as iPSC-derived RPE cells are easy to generate, are available at a high degree of purity, easily develop into highly polar, pigmented monolayers and possess the ability to accomplish many typical RPE-specific functions such as POS phagocytosis and others [8].

## Induced pluripotent stem cell technology

The generation of iPSCs was first reported in 2006 by retrovirally transducing four defined transcription factors (Oct4, Sox2, c-Myc and Klf-4) proving sufficient to reprogram mouse fibroblasts as well as adult human dermal fibroblasts into PSCs [9], [10]. Subsequently, the pluripotency of iPSCs allows their differentiation into various cell types of each germ layer: endoderm (including pancreatic islet cells and esophageal cells), mesoderm (including cardiomyocytes and renal cells) and ectoderm (including the neuroectodermal RPE cells) [11]. Not only is it feasible to differentiate a multitude of different cell types from an individual iPSC stock, iPSCs are well suited to be subjected to genetic manipulation. A most elegant tool was introduced to manipulate the mammalian genome based on the RNA-guided Cas9 enzyme [12]. After Cas9 binding to a so-called protospacer adjacent motif (PAM), hybridization of the 5′ end (spacer) of the single guide RNA molecule to a typically 20-nt complementary sequence (protospacer) activates the Cas9 nuclease domain, leading to cleavage of the target DNA sequence at both strands. This can eventually be used to introduce defined disease-causing mutations or generate knock-out cell lines in isogenic backgrounds to overcome the rather high variability of iPSC-derived cell lines [13], [14], [15].

In this article, we focus on iPSC-RPE cells and their value in ophthalmic research but also the challenges one encounters when modeling monogenic and complex retinal diseases. We exemplify the use of the iPSC and CRISPR/Cas approaches on two well-known degenerative disorders of the RPE, namely the autosomal dominant Best vitelliform macular dystrophy (BVMD), a monogenic disease with early childhood onset, and age-related macular degeneration (AMD), a common complex disease of the elderly populations of Western countries. With these two examples, we want to cover the wide range of effect sizes of genetic variants contributing to disease and how this may or may not be modeled appropriately in cell culture systems. A brief summary of benefits and limitations of the iPSC-derived models is given in Table 1.

**Table 1:**
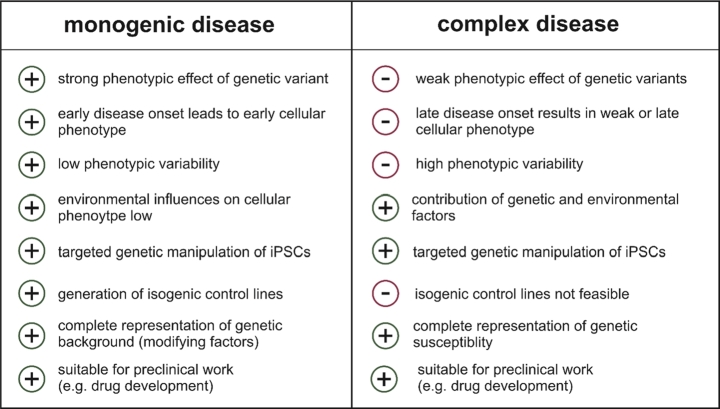
Modeling monogenic and complex age-related diseases via induced pluripotent stem cells (iPSCs).

## Modeling the autosomal dominant BVMD

Mutations in the human *bestrophin-1* (*BEST1*) gene cause a heterogenous group of diseases including the autosomal dominant BVMD (MIM 153700) [16], [17]. BVMD is the most common pathology of the *BEST1*-related retinal dystrophies with an estimated prevalence between 1:5000 and 1:50,000 [18]. To date, over 250 independent disease-causing mutations in *BEST1* have been reported (https://databases.lovd.nl/shared/genes/BEST1, accessed in July, 2021).

**Figure 1:**
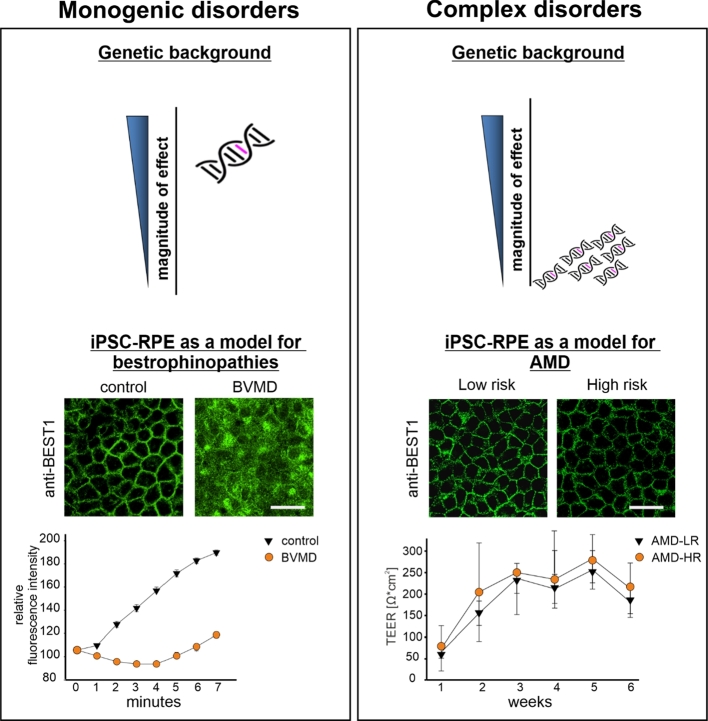
Schematic representation of iPSC-derived model systems for monogenic and complex degenerations of the RPE. In monogenic disorders such as the autosomal dominant Best vitelliform macular dystrophy (BVMD), a single genetic variant confers a strong effect often leading to early onset of the disease. An *in vitro* model of iPSC-RPE cells derived from a healthy control person and a BVMD patient reveals distinct differences between control cells and BVMD cells. In the patient cell line, most of the BEST1 protein is retained in the cytoplasm while functional consequences of disease-associated BVMD variants can be easily recorded by analyzing BEST1 chloride conductance via a halogenide transporter assay (n=6 for each control and BVMD). In contrast, complex disorders such as age-related macular degeneration (AMD) are associated with a great number of independent genetic variants, which individually contribute only a small magnitude of effects. Phenotypically, iPSC-RPE from donors with an exceptionally high genetic AMD risk (HR) may not display a characteristic phenotype when compared to a cell line with an exceptionally low AMD risk profile (LR). Structural changes may not be evident by staining the RPE cells with antibodies of a marker protein (anti-BEST1) or by measuring transepithelial electrical resistance (TEER) over several weeks of culturing the cells, regardless if the cells were exposed to oxidative stress or not (n=4 for each risk group). Scale bar, 20 µm.

*BEST1* encodes the subunits of a homopentameric ${\mathrm{Ca}}^{2+}$-sensitive ${\mathrm{Cl}}^{-}$ channel, which localizes most prominently to the basolateral plasma membrane of the RPE [19]. Pathological mutations affect channel localization, stability and ion gating properties [20], [21], all features resulting in reduced ion transport activity [22], ultimately leading to impaired RPE homeostasis and commonly to central vision loss in BVMD patients. To date, there is no treatment for BVMD or any of the *BEST1*-linked diseases and suitable model systems have been sought for the development of tailored therapies. As the RPE is the primary site of pathology of BEST1-associated diseases, patient-derived iPSC-RPE cell lines have become a focus of research to deepen our understanding of the molecular processes underlying the disease mechanisms. Major advantages of this system include robust expression of the BEST1 protein in the polarized iPSC-RPE cells at the basolateral membrane [8], measurable ${\mathrm{Cl}}^{-}$ currents [23], cellular accumulation of misfolded protein [20] and dysregulated chloride conductance in iPSC-RPE harboring pathogenic *BEST1* variants [21]. iPSC-RPE cells derived from BVMD patients show a robust phenotype clearly distinguished from cells obtained from healthy donors (Figure 1). In addition, the BVMD-associated phenotype can readily be used to test experimental treatments such as small molecule applications and others. Reversing the pathologic phenotype of protein mislocalization and impaired chloride conductance can easily be recorded on high-throughput screening platforms, such as the Operetta CLS™ high-content analysis system for analysis of protein localization [24] or a multiwell plate-based halogenide transporter assay for analysis of BEST1-mediated chloride conductance [25].

## Modeling the complex AMD

AMD is the leading cause of vision loss in developed countries and is the third most common cause globally, after cataract and glaucoma, of blindness [26]. AMD primarily affects the central part of the retina, known as the macula, which has the highest density of cone photoreceptors and is highly specialized for high-resolution and color vision [27], [28]. The risk to develop late-stage AMD is influenced by a combination of genetic and environmental factors, typical of complex disease etiologies. Our understanding of the genetic basis of AMD has greatly been aided by genome-wide association studies (GWAS), the latest of which identified 52 independent single nucleotide polymorphisms (SNPs) in 34 gene loci associated with the disease at genome-wide significance [29]. Aging, cigarette smoking, sunlight exposure, hypertension, cardiovascular disease, alcohol consumption and diet are considered environmental risk factors for AMD [30]. These factors are all associated with increased intracellular oxidative stress, which is possibly a key feature of the molecular pathobiology of AMD (reviewed in [31], [32]). Specifically, there is a high oxidative stress burden in the RPE due to its high metabolic rate, the enormous oxygen tension between the retina and the oxygen-rich choriocapillaris and a situation of increased photooxidation caused by intense light exposure and POS phagocytosis [4], [32]. Other disease mechanisms include dysregulation of the complement cascade and the remodeling of the extracellular matrix (ECM) involving structural changes in Bruch’s membrane, a five-layered ECM between the RPE and the choriocapillaris [33].

A particular demand for a cellular model of a complex disorder like AMD is to mirror the influence of both genetic and environmental factors. A variety of animal models are used, but none of these models fully mimic the multitude of AMD characteristics [34], [35]. Of note, most species do not have a macula with a marked density of cone photoreceptors, a distinctive feature in humans that is particularly vulnerable for diseases such as BVMD and AMD. Also, no animal model available recapitulates the full disease spectrum of AMD, but only specific manifestations of late-stage AMD can be addressed experimentally, for example by laser treatment or increased environmental partial oxygen pressure [36], [37]. Since the genetic basis of AMD is of a heterogenous nature and inherently complex, generation of a genetically engineered mouse model for AMD reflecting an individual genetic background with a high-risk profile for AMD seems not practicable by current technology.

A number of studies reported defined AMD phenotypes for patient-derived iPSC-RPE cells, specifically increased susceptibility to oxidative stress, higher ROS levels upon oxidative stress induction, upregulation of complement genes and mitochondrial dysfunction [38], [39], [40]. Still, the majority of these studies ignore the contribution of the highly complex genetic basis of AMD and mainly focus on a single genetic risk variant, mostly the well-known CFH:Y402H variant in the complement factor H gene [41]. Other studies did not determine the genotypes of their donors at all, disregarding one of the major components underlying AMD etiology [39]. Given the complex genetic architecture of AMD, an ideal model needs to reflect an individual’s own risk profile as accurately as possible. To this end, we have established a comprehensive repository of iPSC lines, for which we selected the donors not by their phenotype, but by the degree of their genetic AMD risk (Figure 1). This is best done by calculating a genetic risk score (GRS) for each person which collectively counts and weights the entirety of the risk-altering alleles of a subject. Subsequently, the power of the GRS approach is strengthened by multiplying the number of risk alleles with the corresponding effect sizes as determined by the corresponding GWAS [29]. Comparison to a reference population allows an individual GRS to be categorized on a scale from a very low (LR, category 1) to a very high (HR, category 5) genetic risk [42]. Choosing donors from the two extreme ends of the genetic AMD risk spectrum, the influence of the genetic contribution to disease between the two groups may become appreciable, specifically in a disease such as AMD with a strong genetic background and an odds ratio between HR and LR exceeding 20 [26]. Even in cell lines from the extreme ends of the AMD risk spectrum, phenotypic manifestations are anticipated to be subtle and thus difficult to define in any experimental setting. To overcome this difficulty inherent to complex diseases, exogenous stressors can be used to enhance phenotypic expression. For example, oxidative stress can be triggered in RPE by exposing the cells to chemical (e. g., sodium iodate or paraquat), physical (e. g., blue light) or physiological (e. g., POS feeding) stressors [43], [44], [45]. In age-related diseases such as AMD, an *in vitro* cellular system should additionally account for increasing age as a major risk factor of the disease. While there are complex diseases which manifest early in life such as autism spectrum disorder, the majority of complex diseases, including AMD, take several decades to manifest pathological changes. It is unclear whether iPSCs are suited to reproduce late phenotypes, as several lines of evidence suggests that iPSC-derived cells still represent rather a stage of very early retinal development [46]. With this in mind, mimicking aging in iPSC-RPE as a model for AMD will be a major challenge that has to be overcome in the future, possibly by exposing differentiated cells to conditions greatly accelerating the aging process.

## Conclusion

iPSC technology has revolutionized research directed at the understanding of biological mechanisms of gene function in health and disease. A major advantage offered by this technology is the possibility to establish cell lines from human patients affected by monogenic but also by complex diseases with the option to enhance phenotypic expression of genetic variants by exposing the cell cultures to environmental stressors of choice. A further benefit of iPSC technology lies in the potential to differentiate the pluripotent cells in almost all cell types of interest, implicitly addressing cell type-specific effects which otherwise may remain undetected. While we contrasted the application of iPSC culture models for two retinal diseases with differing contributions of the genetic spectrum ranging from monogenic to complex inheritance, we have chosen the example of the age-related and complex disease AMD to direct our attention to the downside of iPSC-derived cells, namely the early developmental age of such cells. This could greatly hamper the suitability of iPSC-derived models to investigate age-related diseases, unless ways can be delineated to define conditions that transform the cells into a state of advanced age and rather chronic disease exposure. Then, such models will be broadly applicable, specifically as a platform for understanding and treating the frequent aging diseases of humankind.
